# Sources and applications of endothelial seed cells: a review

**DOI:** 10.1186/s13287-024-03773-6

**Published:** 2024-06-18

**Authors:** Dan Deng, Yu Zhang, Bo Tang, Zhihui Zhang

**Affiliations:** 1 Department of Cardiovascular Medicine, Center for Circadian Metabolism and Cardiovascular Disease, Southwest Hospital, Army Medical University, Chongqing, China; 2 Chongqing International Institute for Immunology, Chongqing, China

**Keywords:** Endothelial cells, Vascular diseases, Regenerative medicine, Seed cells, Vascularization

## Abstract

Endothelial cells (ECs) are widely used as donor cells in tissue engineering, organoid vascularization, and in vitro microvascular model development. ECs are invaluable tools for disease modeling and drug screening in fundamental research. When treating ischemic diseases, EC engraftment facilitates the restoration of damaged blood vessels, enhancing therapeutic outcomes. This article presents a comprehensive overview of the current sources of ECs, which encompass stem/progenitor cells, primary ECs, cell lineage conversion, and ECs derived from other cellular sources, provides insights into their characteristics, potential applications, discusses challenges, and explores strategies to mitigate these issues. The primary aim is to serve as a reference for selecting suitable EC sources for preclinical research and promote the translation of basic research into clinical applications.

**Graphical abstract:**

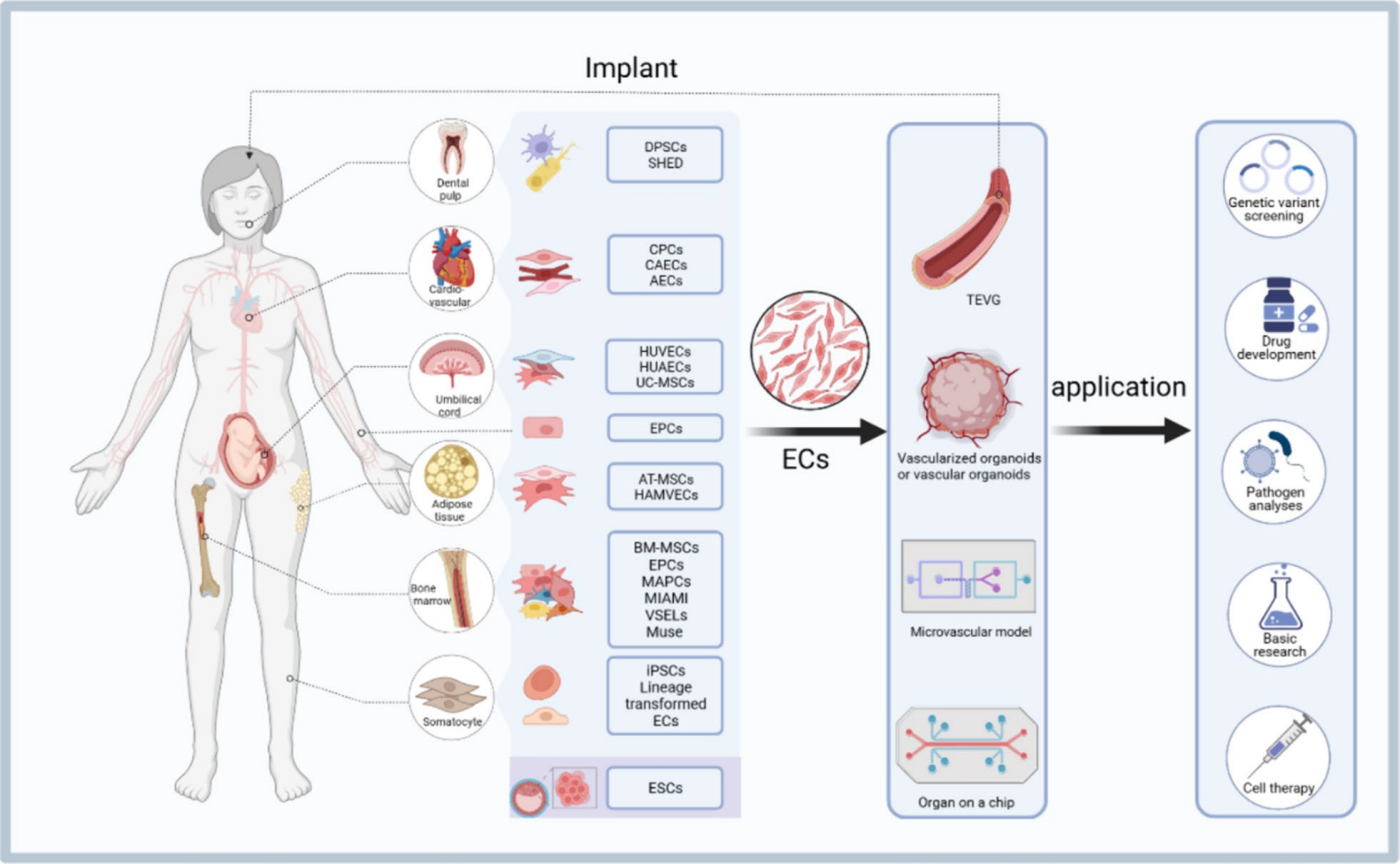

## Background

**Fig. 1:**
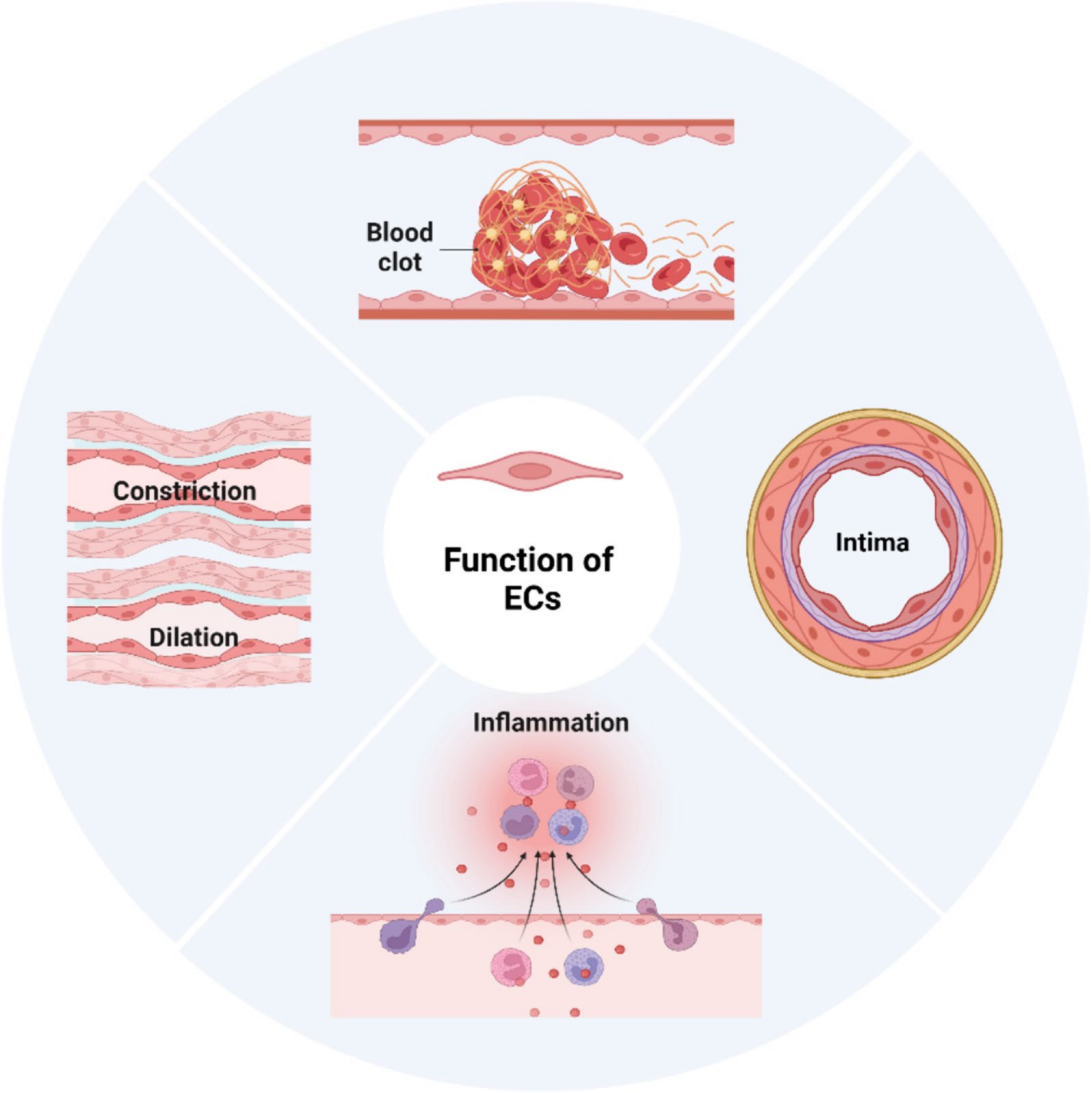
Brief summary of EC functions. The functions include vascular wall formation, regulation of vascular permeability, inflammatory response mediation, thrombus formation inhibition, chemical signal secretion, regulation of vascular dilation and constriction. Figure created using BioRender.com

Endothelial cells (ECs) form a monolayer of flattened epithelial cells and are predominantly located on the inner luminal surface of blood vessels. ECs are widely distributed throughout the vascular system, which encompasses arteries, veins, and capillaries. ECs are pivotal as a hemostatic barrier, mediating blood–tissue interactions and securely maintaining vascular permeability and integrity through tight intercellular junctions. ECs actively secrete multifunctional bioactive molecules, including nitric oxide, angiotensin, prostaglandins, and thromboxanes, orchestrating processes such as vascular constriction, dilation, coagulation factor regulation, and immune response modulation. Furthermore, ECs regulate the metabolic homeostasis of the body’s three major nutrients—carbohydrates, proteins, and fats—thereby ensuring metabolic balance (Fig. 1).

Researchers have extensively used the indispensable biological functions of ECs as seed cells for tissue-engineered vascular graft (TEVG) endothelialization and organoid model vascularization in preclinical investigations. Vascular transplantation is an effective approach for treating vascular diseases. Graft failure is primarily caused by inadequate endothelialization within the implanted scaffold, leading to thrombus formation and luminal obstruction. Consequently, deliberately integrating ECs significantly enhances vascular graft patency through augmented endothelialization [1]. Clinically relevant organoids undergo increased apoptosis as their volume increases primarily due to hypoxia and the accumulation of metabolic byproducts [2]. Introducing ECs to facilitate organoid vascularization improved blood perfusion and promoted more robust viability [2].

ECs are crucial in disease pathophysiology, including cardiovascular disorders, diabetes, chronic renal failure, and various tumors. These diseases can be influenced by specific pharmacological agents, such as antitumor agents, immunosuppressants, and growth factors, which modulate EC proliferation and migration. Consequently, they affect crucial processes such as angiogenesis and vascular repair. Harnessing the potential of ECs to establish in vitro disease models and drug screening platforms would enable the investigation of the underlying mechanisms of these diseases, conduct drug toxicity assessments, and foster the development of novel therapeutic agents in a controlled experimental environment. Several therapeutic approaches using endothelial progenitor cells (EPCs), particularly those enriched for the CD34 + and CD133 + phenotypes, have progressed to clinical trials [3]. These cell-based interventions have demonstrated promising effects in ameliorating angina symptoms, enhancing exercise tolerance, improving left ventricular function, and augmenting myocardial perfusion in patients with refractory angina, highlighting their potential in regenerative cardiovascular therapies.

Numerous EC sources are available for in vitro vascularization and laboratory research, and include stem cell-derived and primary cells. Nonetheless, a comprehensive summary and comparison of these cell sources is lacking. In this review, we attempt to present a comprehensive overview of the diverse sources of ECs, considering their applications, advantages, and disadvantages (Table 1). We aim to provide a reference to aid researchers in selecting the most appropriate EC sources for preclinical investigations, considering their specific research contexts.

**Table 1:** Brief summarization of different sources of ECs and their advantages, disadvantages and applications

Source	Advantages	Disadvantages	Main applications
ESCs	High differentiation potential	Limited sources	Disease model establishment
	Strong regenerative capacity	Higher immunogenicity	Drug screening platform
	High plasticity	High research cost	Vascular graft endothelialization
		Technical challenges	Organoid vascularization
		Tumorigenicity	Cell therapy
hiPSCs	Accessible in adults	High research cost	Patient-specific disease research
	Ethical concern-free	Significant technical challenges	Disease model establishment
	No or extremely low immunogenicity	Potential tumorigenicity	Drug screening platform
	Patient-specific		Vascular graft vascularization
	Infinite sources		Organoid vascularization
	High plasticity		Cell therapy
MSCs	Readily accessible in adults	Limited differentiation capacity	Cell therapy
	Wide range of sources	Short In vitro survival time	
	Easy to isolate and expand	Subject to individual health status	
	Low immunogenicity		
EPSs	Readily obtainable in adults	Challenging to Isolate	Cell therapy
		Limited quantity	Vascular graft endothelialization
		Heterogeneity	
		Lack standardized culture conditions	
		Subject to individual health status	
CPCs	Readily obtainable in adults	Difficult to isolate	Cell therapy
		Limited quantity	
		Heterogeneity	
		Inconsistent in vitro culture	
		Subject to individual health status	
HUVECs	Easy to isolate and culture	Limited source	Disease model establishment
	Stable biological characteristics	Poor plasticity	Drug screening platform
	Low immunogenicity		Vascular graft endothelialization
			Organoid vascularization
Other primary ECs	Readily obtainable in adults	Heterogeneity	Patient-specific disease research
	Widely sourced	Limited plasticity	Establishment of disease models
		Finite lifespan	Drug screening platform
			Transplanted blood vessel vascularization
			Organoid vascularization
Lineage conversion	Readily obtainable in adults	High research costs	Disease model establishment
	Low immunogenicity	Significant technical challenges	Drug screening platform
	Strong plasticity	Influenced by Individual health status	Cell therapy
	Low risk of tumorigenicity		
Subpopulations of bone marrow stromal cells	Readily obtainable in adults	Limited research	Cell therapy
	Abundant sources	Unclear mechanisms of differentiation	
	Strong differentiation potential		
Dental pulp	Readily obtainable in adults	Limited research	Investigation of fate determination in dental tissue vascular formation

## Stem-cell derived ECs

### Embryonic stem cells (ESCs)

A significant scientific milestone was achieved in 1998 when researchers successfully isolated ESCs from human blastocyst-stage embryos. This groundbreaking discovery unveiled the remarkable capacity of ESCs to differentiate into a wide array of cell types, including ECs [4]. Years of rigorous investigation established methodologies for ESC differentiation into ECs and are now widely used in scientific research.

The prominent ESC lines in use are the H1 and H9 lines. The induction strategies predominantly involve monolayer-directed differentiation into ECs and three-dimensional (3D) cultivation using embryoid bodies (EBs). Additionally, certain laboratories co-culture ESCs with stromal cells such as S17 mouse bone marrow (BM) cells, OP9 cells, and M2-10B4 cells to induce EC differentiation. Co-culture recreates a microenvironment that guides stem cell differentiation while avoiding the complexity of EBs. Nevertheless, co-culture may introduce xenogeneic elements, raising concerns about potential unknown biological contamination and immune rejection risk. Despite ongoing debates regarding the optimal method, each co-culture method successfully directed ESC differentiation into ECs with specific functional attributes. For example, human ESC-derived ECs (ESC-ECs) cultured through monolayer-directed differentiation produced nitric oxide, responded to chemical stimuli, and supported the development of new blood vessels, ultimately restarting blood flow in ischemic mouse limbs [5]. Shen et al. illustrated the potential of mouse ESC-ECs derived from EB formation for engineering blood vessels. That seminal study marked the initial evidence of ESCs as viable seed cells in tissue-engineered vascularization [6]. Furthermore, ESC-ECs represent a valuable cellular source for inducing vascular regeneration in ischemic myocardium tissue. When ESC-ECs cultured on fibronectin-coated dishes were introduced into the hearts of mice with ligated left anterior descending coronary arteries, microcapillaries and small veins were notably increased within the infarcted region, leading to improved functionality of the infarcted heart [7]. Moreover, ESC-ECs have been used under specific chemical conditions to construct versatile in vitro 3D vascular models [8] and were pivotal in establishing intricate vasculature-like networks in organoid systems [9–11]. These advancements have contributed significantly to the important preclinical research foundation required for drug screening, disease modeling, and related in vitro studies.

While ESC-ECs hold great promise for research, several issues should be considered: (1) the use and destruction of human embryos in ESC research result in religious and ethical concerns [12]. Recent Chinese policies have reinforced the alignment of ethical principles with social development, broadening the scope of patent protection for human ESC (hESC) research outcomes. This reinforcement has greatly encouraged technological advancements in this domain, fostering a conducive environment for innovation and discovery [13, 14]. (2) several readily available ESC lines are obtained through isolation and subsequent in vitro proliferation, often involving exposure to animal-derived components. This approach carries potential risks associated with toxic substances, microorganisms, and unforeseen biological contaminants, which may elicit immune responses. The strategies to address this issue include developing serum substitutes and culture medium free from harmful xenobiotics [15]. Additionally, implementing feeder-free culture systems is aimed at minimizing contamination from xenogeneic sources [16]; (3) transferring ESCs from embryos to culture dishes can lead to progressive adaptation to the new environmental conditions. This adaptation may result in alterations, including nuclear structure changes, DNA damage, double-strand DNA cleavage and fragmentation, loss of heterozygosity, increased cancer-related gene diversity [17], and the potential for mutations during extended cultivation periods [18]. Nevertheless, ESCs maintain a higher degree of genomic stability than somatic cells [19]. Genetic instability during ESC in vitro culture can be substantially reduced by optimizing cultivation conditions, following standardized protocols, minimizing passages, regular karyotyping, and vigilantly monitoring genomic stability. These strategies collectively aid the preservation of ESC integrity and reliability for research and potential clinical applications. (4) Somatic cell nuclear transfer (SCNT)-derived cells present a promising solution to immune rejection in transplantation, as they can be customized to match the patient’s genetic composition. Transferring the nucleus of a patient’s somatic cell into an enucleated oocyte creates ESCs (NT-ESCs) genetically identical to the patient. This method was successful in primates and is being adapted for use with human cells to produce stem cells compatible with the patient’s immune system for therapeutic purposes [20, 21].

### Human induced pluripotent stem cells (hiPSCs)

The emergence of hiPSC technology has produced patient-specific hiPSCs free from ethical and immunological concerns [22]. hiPSC-derived ECs (hiPSC-ECs) that underwent CD144 antibody-conjugated magnetic bead sorting exhibited typical EC morphological and functional characteristics, including tubular structure formation, responsiveness to inflammatory signals, and nitric oxide production [23]. Notably, the hiPSC-ECs demonstrated an angiogenesis and endothelial differentiation capacity comparable to that of their ESC-derived counterparts. This capacity positions hiPSC-ECs as a valuable platform for disease modeling and drug screening [24].

The primary application of hiPSC-ECs is their direct use as a substitute for injured or dysfunctional ECs to facilitate impaired vascular system restoration. Transplanting hiPSC-ECs into an animal model of ischemia promoted the repair of injured tissues and enhanced tissue perfusion [25]. However, there is no definitive conclusion on the angiogenic capacity between hiPSC-ECs and human umbilical vein ECs (HUVECs). One study reported that hiPSC-ECs have a greater capacity to integrate into the developing zebrafish vascular system than HUVECs [26]. Conversely, other studies indicated that while hiPSC-ECs formed perfused vessels on the dorsal side of mice, they exhibited a reduced density and quantity of perfused vessels compared to HUVECs, resulting in diminished vessel maturation [27]. Nonetheless, these results collectively confirm the capacity of hiPSC-ECs to directly promote blood vessel formation in vivo and enhance blood perfusion for treating ischemic disorders.

hiPSC-ECs are pivotal in vascular engineering and organ transplantation. hiPSC-ECs are an abundant source of ECs and have wide-ranging applications in bioprinting [28], microfluidic devices [29], organ-on-chip technologies [30, 31], and organoid vascularization [32, 33]. Furthermore, hiPSC-ECs are a simplified yet potent platform for developing therapeutic interventions, enabling the accurate replication of human physiology that facilitates precise disease modeling and tissue regeneration.

The blood vessel organoids produced from hiPSC-ECs exhibited an impressive resemblance to the structure and function of native human blood vessels [34]. hiPSC-ECs implanted into decellularized vascular scaffolds demonstrated robust adhesion, stability, permeability, and sustained vascular patency [35]. Recent breakthroughs have emerged in utilizing hiPSC-ECs for constructing small-caliber TEVGs, where the implantation of these grafts in nude mice for 30 days revealed no noticeable thrombus formation, while non-endothelialized grafts exhibited significant thrombus formation [36]. These developments form a critical research foundation for developing innovative allogeneic vascular grafts for patients with vascular diseases.

Disease-specific hiPSC-ECs are a promising avenue for investigating differential gene expression. Using disease-specific hiPSCs avoids the constraints associated with gene editing and other requirements for disease modeling linked to ESCs. Disease-specific hiPSC-ECs have been successfully derived from patients with diabetes [37], peripheral artery disease [38], end-stage renal disease [39], type A hemophilia [40], and pulmonary arterial hypertension [41]. These disease-specific hiPSC-ECs facilitated the establishment of in vitro disease models with impaired endothelial function, providing crucial insights for developing therapeutic strategies based on autologous cells. Additionally, hiPSC-ECs from patients with genetic disorders and rare diseases exhibit disease-relevant phenotypic traits, providing vital preclinical data to clarify disease mechanisms and explore potential therapeutic approaches [42–45].

hiPSCs exhibit exceptional pluripotency compared to other cell types. hiPSCs exposed to specific tissues generated tissue-specific microvascular ECs (MVECs). Notably, hiPSC-derived brain MVECs could be integrated with organ-on-chip technology to create sophisticated neurovascular units [46]. This innovation enables the study of the blood–brain barrier response to relevant diseases and drug screening in the central nervous system [47, 48]. In a co-differentiation system involving hiPSC-ECs and hiPSC-derived cardiomyocytes, the hiPSC-ECs demonstrated cardiac marker expression similar to that observed in primary cardiac microvasculature [49]. This distinct characteristic confers hiPSC-ECs with the capacity to faithfully replicate the authentic biological state of ECs across multiple organs.

While reprogramming hiPSCs from patient or donor somatic cells is relatively straightforward, hiPSC-ECs have limited practical application in autologous cell therapy for elderly people or patients with genetic diseases. Gene editing technologies such as CRISPR can correct gene diseases in hiPSCs and efficiently model gene mutations [50–52]. Furthermore, hiPSCs carry the risk of tumorigenicity during differentiation. Accordingly, the evaluation of hiPSC-EC safety and stability can be facilitated by extending the culture duration to mimic senescence and by conducting in vivo tumorigenicity tests. Notably, even autologous hiPSCs exhibit a low immunogenicity profile [53]. Nevertheless, this concern can be addressed using human-derived components [54] or serum-free cultivation strategies [55] to avoid the use of xenogeneic materials in generating xeno-free (XF)-hiPSC-ECs.

### Mesenchymal stem cells (MSCs)

MSCs represent a diverse subset of stromal stem cells with multipotent differentiation capacity that can be isolated from various human tissues, such as BM, adipose tissue (AT), umbilical cord (UC), UC blood, placenta, dental pulp (DP), and amniotic fluid [56]. MSCs from different anatomical sources exhibit a distinct potential for endothelial differentiation. For example, BM-MSCs isolated through density gradient centrifugation and stimulated with growth factors differentiated into ECs and formed capillary-like networks on matrix gels [57].

Laboratory research principally explores the therapeutic potential of MSC-ECs for ischemic diseases. For example, MSCs underwent differentiation into smooth muscle cells (SMCs) and ECs in a canine model of chronic ischemia, promoting angiogenesis and enhancing cardiac function [58]. Similarly, introducing MSCs into the endocardium in a porcine model of chronic myocardial infarction led to SMC and EC differentiation in the infarcted and border regions, facilitating the formation of blood vessels of varying sizes [59].

While MSCs are ubiquitously sourced, they exhibit heterogenous cellular morphology, surface marker expression, and differentiation potential, resulting in diverse biological characteristics across varying origins and batches. Furthermore, endothelial differentiation is typically subject to inefficiency and impurity. Specific growth factor cocktails, small molecules, or biomaterial scaffolds can augment differentiation efficacy and cell purity. Complementarily, advanced cell sorting techniques and functional assays ensure a homogenous and functional population of derived ECs.

## Adult progenitor cell-derived ECs

### Endothelial progenitor cells (EPCs)

In 1997, Asahara et al. [60] discovered circulating EPCs in peripheral blood, which were subsequently found to contribute significantly to vessel formation in murine and rabbit hindlimb ischemia models. These EPCs were characterized as a specific population of progenitor cells expressing markers such as CD133, CD34, and KDR. EPCs are EC precursor cells and are critical in angiogenesis and vasculogenesis [61]. EPCs are present in peripheral blood and have been identified in UC and BM. It was widely accepted that circulating EPCs originate from the BM. However, recent research using advanced techniques such as single-cell RNA sequencing, in vivo genetic tracing in murine models [62], and the analysis of ECs in the blood of allogeneic BM transplantation patients established that circulating EPCs do not originate from the BM [63]. Instead, they reside within the blood vessel walls.

TEVGs constructed using EPCs exhibit prolonged patency and demonstrate vascular contractile and relaxation characteristics similar to natural arteries [64]. Additionally, EPCs can swiftly coat implanted artificial vascular scaffolds, offering crucial support for in situ endothelialization induction [65]. Similar to MSCs, EPCs are also used in cell therapy for ischemic conditions. In vitro expanded EPCs enhanced cardiac function recovery, increased capillary density, reduced left ventricular scar formation, and facilitated vascular neogenesis in rat models of myocardial infarction [66]. Furthermore, the cells exhibited potential in ameliorating myocardial fibrosis, improving cardiac function in rat models with coronary microcirculatory disorders [67]. Combined transplantation is an alternative to the exclusive use of EPCs for therapeutic purposes. Co-transplanting EPCs and MSCs in laboratory settings, as opposed to EPCs alone, significantly enhanced organ function and fostered tissue regeneration and functional repair in ischemic diseases, albeit without significant differences in angiogenesis [68]. Therefore, the combined transplantation of EPCs and other stem cells holds potential advantages in tissue engineering. However, there is a pressing need for thorough foundational research to assess the feasibility of this approach before clinical trials can be initiated.

The isolation of EPCs from adult sources is hindered by their limited availability and challenges regarding isolation. Moreover, the discernible heterogeneity of EPCs, stemming from inconsistent culture conditions and a lack of standardization, has contributed to inconsistent experimental outcomes. These factors impose significant limitations on the widespread applicability of EPCs. Using more efficient and specific markers with advanced sorting technologies such as flow cytometry with multiple surface antigens (CD34 + , CD133 + , VEGFR2 +) enhances the purity and yield of the isolated EPCs. Establishing a unified, validated culture system and characterization criteria for EPCs fosters their self-renewal and preservation of characteristics, augmenting research outcome consistency and comparability.

### Cardiac progenitor cells (CPCs)

In 2001, Orlic et al. successfully isolated c-Kit + cells from BM, which could differentiate into ECs and SMCs when exposed to cytokine mobilization. The CPCs subsequently migrated to the infarcted myocardium, contributing to myocardial cell regeneration [69]. Subsequent experiments validated the presence of CPCs in the heart. CPCs could differentiate into myocardial cells, SMCs, and ECs during cultivation. Additionally, CPCs were pivotal in promoting neovascularization within infarcted myocardium and facilitating cardiac function restoration. This groundbreaking discovery challenged the traditional notion that myocardial cells are terminally differentiated cells [70].

Various CPCs, including c-Kit + , Sca-1 + , Islet-1 + , cardiac side population, cardiospheres, and cardiosphere-derived cells, have been identified and extensively studied after isolation from the adult heart. In vivo, injected CPCs targeted the myocardial injury site and underwent in situ differentiation into cardiomyocytes, ECs, and SMCs [71, 72]. The intracoronary administration of c-Kit + CPCs in a rat model of acute myocardial infarction resulted in their division and differentiation into ECs and SMCs, effectively reducing the extent of myocardial infarction and enhancing cardiac function [73]. CPCs can be obtained from donor heart biopsies and cryopreserved cardiac tissues, and expanded and differentiated into ECs in vitro [74]. Nevertheless, CPC-ECs demonstrate limited utility compared to ECs derived from alternative sources in disease modeling and vascular engineering, suggesting a lack of superiority.

However, CPC acquisition is challenging due to their restricted availability. Additionally, cellular senescence and genetic mutations may arise during expansion and cultivation, affecting CPC quality and functionality. The complex task of characterizing and identifying CPCs is complicated by their phenotypic heterogeneity, which hinders the definition of specific markers for their isolation and purification. Hence, addressing these challenges and refining the procedures involved in CPC acquisition and cultivation are imperative to develop their therapeutic potential in regenerative medicine [75].

## Primary ECs

### HUVECs

In 1973, Jaffe et al. successfully isolated ECs from human umbilical veins and characterized them using morphological, immunohistochemical, and serological criteria [76]. Since then, HUVECs have been widely used by the global vascular biology community as they are easily obtained, their supply is abundant, and they are cost-effective. Importantly, the wealth of knowledge accumulated over the past five decades has firmly established HUVECs as the preferred source of ECs for scientific research. Additionally, HUVECs are the benchmark for developing innovative approaches in vascular biology and angiogenesis.

In 2004, Koike et al. [77] successfully co-cultured HUVECs with MSCs, constructing a vascular network in vitro. Upon transplantation into mice, this construct demonstrated a sustained presence for up to 1 year. Subsequently, as tissue engineering gained prominence, HUVECs have been extensively used as a source of ECs for tissue-engineered construct endothelialization [78, 79], vascular chip fabrication [80], bioink preparation [81], and various other applications. Data from 2013 to 2018 indicate that 59% of studies used HUVECs as the primary cell source for ECs [82]. Furthermore, HUVECs are used in organoid vascularization, aiding the development of functional blood vessel networks within these artificial organ-like structures. For example, Shi et al. co-cultured HUVECs with hESCs or hiPSCs, leading to their directed differentiation into vascularized brain organoids. HUVECs interconnected within the brain organoids formed a complex, permeable vascular system that persisted for > 200 days. Upon transplantation into animal models, HUVECs integrated with the host murine vascular ECs within the brain organoid, establishing a functional vascular network system characterized by blood flow. This integrated vascular system demonstrated the maturity and viability of the engrafted brain organoid [83]. Additionally, Takebe et al. co-cultured hiPSC-derived hepatic endoderm cells with HUVECs and BM-BMCs, inducing the formation of liver buds (hiPSC-LBs) comprising 3D spherical tissues. The hiPSC-LBs were transplanted into immunodeficient mice and generated intricate vascular networks within 48 h that integrated with the host vasculature. Real-time imaging confirmed the perfusion of host blood, validating the establishment of a functional human vascular network [84].

However, the application of HUVECs is subject to shortcomings. First, the inherent heterogeneity and tissue-specific nature of ECs in normal organs and tissues means that using HUVECs or other primary EC lines to generate vascular systems within organoids may not offer the same advantages as using PSC-derived ECs, which exhibit greater plasticity. Second, HUVECs have a limited lifespan, necessitating recurrent cell acquisition from fresh donors. Lastly, prolonged HUVEC culture may result in the loss of their native characteristics and functionality. Co-culturing HUVECs with ECs from other sources, such as specific adult or fetal tissues, and using 3D culture systems or bio-printing techniques to create microenvironments can better mimic in vivo conditions, enhancing the maintenance of HUVEC native morphology and function.

### Other primary ECs

No discernable distinctions have been observed in cell proliferation, metabolic activity, membrane integrity, and vasoactive substance production between human umbilical artery-derived ECs (HUAECs) and HUVECs [85]. Consequently, some research laboratories use HUAECs as a source of ECs for in vitro investigations [86–88]. Primary ECs are also widely derived from various human tissues, extending beyond UC origins. MVECs isolated from the retina [89], AECs [90], coronary artery ECs [91], brain MVECs [92], and lung MVECs [93] have been used to promote vascularization in engineered tissues and organoids. Notably, several laboratories have devised methodologies for isolating MVECs from AT, which is abundant and accessible. However, differentiated MVECs constitute a heterogeneous amalgamation encompassing subpopulations with arterial, venous, and lymphatic lineages. Consequently, additional research is warranted to clarify the phenotypic heterogeneity of MVECs [94, 95]. These primary cells are a potential invaluable source for patient-specific cell therapies and in vitro disease modeling. However, their limited lifespan and relatively restricted adaptability limit their application. Currently, no compelling evidence supports their distinct advantage over HUVECs.

### Lineage conversion ECs

Recent cellular reprogramming advancements have introduced a novel approach for converting one somatic cell type into another, bypassing the pluripotent state. This method involves inducing functional cells from a specific lineage through the exclusive use of lineage-restricted transcription factors, offering a promising means of generating functional ECs. Significantly, this strategy avoids the potential tumorigenic risks associated with PSC-derived ECs.

ETV2 is a pivotal factor in EC lineage regulation and reprogramming [96]. The transient expression of ETV2 enables the reprogramming of amniotic cells [97, 98] and human fibroblasts [99–101] into functional ECs without undergoing a pluripotent transition. These reprogrammed cells harbor the potential to establish mature and functional vascular systems in vivo, promoting blood flow restoration in ischemic limbs. Palikuqi et al. [102] “reset” mature human ECs into adaptive angiogenic cells through the transient expression of ETV2, which confers plasticity upon the ECs, allowing them to adapt to novel environments. Beyond ETV2, transducing Oct4, Klf4 [103] or Foxo1, Er71, Klf2, Tal1, and Lmo2 [104] into human fibroblasts induced their differentiation into functional ECs. Sayed et al. reported that innate immune signaling activation, achieved through the synergistic action of small molecules and Toll-like receptor 3 agonists combined with endothelial growth factors, successfully initiated human fibroblast transdifferentiation into ECs [105].

The donors’ age and health status can influence the state of reprogrammed ECs, encouraging efforts to leverage cell-regulating factors to enhance reprogramming.

## Other cell-derived ECs

### BM stromal cell subpopulations

BM-derived multipotent adult progenitor cells (MAPCs) can differentiate into ECs. MAPCs promoted angiogenesis through endothelial activation in a murine hindlimb ischemia model [106, 107]. While MAPCs and MSCs can be extracted from BM, multiple studies have conclusively demonstrated that MAPCs have significantly greater differentiation potential than MSCs [108, 109]. For example, MSC-like cells lacking Oct4 expression exhibit limited differentiation capabilities towards ECs and hepatocyte-like cells.

D’Ippolito et al. isolated a population of adult multipotent cells, termed marrow-isolated adult multilineage (MIAMI) cells, from the BM [110]. In vitro experiments established that the MIAMI cells could differentiate into CD31 + KDR + vWF + endothelial-like cells, which could form vascular network structures when cultured [111]. Furthermore, transplanting these cells into a murine hindlimb ischemia model facilitated the restoration of the ischemic region [112].

Kucia et al. [113] identified a population of very small embryonic-like stem cells (VSELs) in a subset of BM cells. Subsequently, at least 20 independent research groups confirmed the pluripotent differentiation potential of VSELs across germ layers. For example, transplanting human VSELs into an ischemic model demonstrated the emergence of CD31 + endothelial phenotypes [114]. Additionally, VSEL-like stem cells have been discovered in UC tissues [115]. However, no studies have demonstrated their capacity to regenerate 3D fully functional tissue structures or form teratomas in immunocompromised mice. Therefore, further experimental investigations are required to explore VSEL pluripotency [116].

In 2010, Kuroda et al. demonstrated that adult MSCs under in vitro stress conditions can undergo transformation, exhibiting pluripotent-like characteristics. They referred to these cells as Muse (multilineage differentiating stress-enduring) cells [117]. Following intravenous injection into a murine model of aortic aneurysm, Muse cells spontaneously differentiate into vascular SMCs and ECs, preserving the elastic fibers and attenuating aneurysm expansion [118]. Muse cells are believed to be abundantly distributed in the connective tissues of various human organs, demonstrating the capacity to differentiate into all three germ layers. Upon transplantation, Muse cells preferentially home in to injury sites and spontaneously differentiate into tissue-compatible cells. Due to their non-tumorigenic properties and exceptional homing capabilities, Muse cells are considered to have superior therapeutic potential compared to other types of stem cells [119, 120]. Despite their promising attributes, scientific understanding of Muse cells and their biological characteristics remains relatively limited, necessitating further exploration.

### DP-derived ECs

Marchionni et al. successfully induced endothelial differentiation in DP stem cells (DPSCs) through exposure to VEGF. The induced DPSCs expressed Flt-1 and KDR and created tubular structures on a matrix [121]. Sakai et al. comprehensively investigated the differentiation potential of stem cells from human exfoliated deciduous teeth (SHED). Their work highlighted the successful differentiation of SHED into odontoblasts and ECs. Furthermore, SHED expressed key vascular markers through induction, including VEGFR2, CD31, and VE-cadherin, and effectively organized into functional capillary-like structures [122]. Nevertheless, the initial strategy for endothelial lineage differentiation exhibited remarkably low efficacy. To address this challenge, Gong et al. introduced an innovative approach that used the decellularized extracellular matrix from HUVECs. This bioactive substrate provided a favorable microenvironment for promoting SHED endothelial differentiation. Consequently, the mRNA expression levels of the endothelial-specific markers, CD31 and VEGFR-2, were significantly upregulated within the SHED population [123].

Overexpressing ETV2 in DPSCs promoted EC differentiation [124]. In the presence of small molecular compounds within the culture system, stem cells from apical papilla differentiated into EC-like cells while maintaining the tubular structure for a more extended period compared to the method proposed by Gong et al. [125]. However, the specific mechanisms underlying this phenomenon remain unexplored.

### EC heterogeneity

Single-cell genomics and in vivo genetic labeling techniques have revealed the nuanced nature of ECs, which demonstrate organ specificity and heterogeneity during their developmental processes [126]. Different induction protocols or stages can yield diverse phenotypic traits within ECs. Consequently, each EC subgroup may be relevant in distinct research endeavors. For example, stem cells promoting angiogenesis, exemplified by stalk cells and tip cells, are ideal for exploring microvascularization in angiogenesis and tissue engineering. In contrast, phalanx ECs, characterized by weaker proliferative and migratory abilities, are more suitable for investigating arterial atherosclerosis and endothelialization in small-diameter vascular grafts [127]. Meticulous selection of the appropriate culture protocols and specific EC phenotypes for foundational research would enable the close replication of physiological or pathological conditions and yield reliable preclinical data.

### Comparative characterization of ECs from various sources

Stem cell-derived ECs, notably iPSC-ECs and ESCs-ECs, exhibit robust proliferative capacity suitable for large-scale production and study, although iPSC-ECs demonstrate marginally reduced expression of key endothelial markers and shear-responsive genes compared to primary ECs [128]. iPSC-EC, ESCs-EC, and MSC proliferation, migration, and angiogenesis significantly outperform that of AECs [129, 130], yet the limited endothelial differentiation potential of MSCs renders them less ideal for vascular engineering. While iPSC-ECs form vessels with lower density and maturity than HUVECs in vasculogenesis [27, 131], they demonstrate excellent endothelial barrier function and responsiveness to shear stress [132].

Adult progenitor-derived ECs are hampered by restricted proliferation and differentiation abilities that may deteriorate with age [133]. Consequently, they are less effective in constructing vessels than HUVECs [134], primarily used in ischemic therapies in animal models.

Directly converting adult cells into ECs bypasses the pluripotent stage, simplifying the process and potentially offering better genetic stability with reduced mutation risks. However, this approach is subject to low conversion efficiency and functionality discrepancies with primary ECs, necessitating further conversion protocol optimization.

## Conclusion and future perspectives

ECs are pivotal in the repair and regeneration of damaged tissues, demonstrating substantial potential in regenerative medicine. Investigating the origin of ECs is extremely important. This review examined the diverse origins of ECs used in preclinical research for clinical applications. HUVECs remain the predominant choice for in vitro EC research. However, the limited lifespan of HUVECs and primary ECs during cultivation and the potential loss of their phenotypic characteristics, suggest that more adaptable ECs from PSCs or progenitor cells may gradually supplant HUVECs. Alternatively, HUVECs could act exclusively as a reference standard for innovative vascular construction methods.

hESC-EC research has increased annually, and their application in basic research will become even more widespread following policy and regulation maturation. Contrastingly, hiPSCs present an unlimited cell source without harming human embryos while preserving the patient’s original disease-specific characteristics. Recent substantial progress has been made in using hiPSC-ECs for disease modeling and engineering tissue vascularization. Specifically, disease-specific iPSC-ECs can closely mimic vascular function and pathological features under disease conditions and will be predominantly used to provide innovative solutions to rare diseases or genetic vascular disorders, and used as drug screening platforms.

MSCs, EPCs, and CPCs exhibit immense potential in cell therapy for ischemic diseases. Their future applications will focus on providing effective support for recovery post-myocardial infarction, reducing the risk of heart failure.

Notably, attention to specific BM stromal cell subpopulations has increased. The capacity for differentiating into ECs and the broad clinical prospects thereof are currently in an intensive exploration phase. This field holds immense potential, heralding significant breakthroughs in regenerative medicine and disease therapeutics.

In summary, a comprehensive understanding of EC origins and characteristics is pivotal for the precise selection of regenerative medicine research models. This understanding would effectively facilitate the translation of research findings into clinical applications, expediting the development of personalized medicine and tissue repair technologies.

## Abbreviations

3D: Three-dimensional
AECs: Aortic endothelial cells
AT: Adipose tissue
CPCs: Cardiac progenitor cells
BM: Bone marrow
DP: Dental pulp
DPSCs: Dental pulp stem cells
EB: Embryoid bodies
ECs: Endothelial cells
EPCs: Endothelial progenitor cells
ESCs: Embryonic stem cells
ESC-ECs: Embryonic stem cell-derived endothelial cells
hiPSCs: Human induced pluripotent stem cells
hiPSC-ECs: Human induced pluripotent stem cell-derived endothelial cells
hiPSC-LBs: Human induced pluripotent stem cell-induced liver buds
HUAECs: Human umbilical artery-derived endothelial cells
HUVECs: Human umbilical vein endothelial cells
MAPCs: Multipotent adult progenitor cells
MIAMI cells: Marrow-isolated adult multilineage cells
MSCs: Mesenchymal stem cells
Muse cells: Multilineage differentiating stress-enduring cells
MVECs: Microvascular endothelial cells
SHED: Stem cells from human exfoliated deciduous teeth
SMCs: Smooth muscle cells
TVEG: Tissue engineered vascular graft
UC: Umbilical cord
VSELs: Very small embryonic-like stem cells
